# Central and peripheral dysmyelination in a 3‐year‐old girl with ring chromosome 18

**DOI:** 10.1002/ccr3.2426

**Published:** 2019-09-27

**Authors:** Dawn Brianna Lammert, David Miedema, Josiree Ochotorena, Nienke Dosa, Kalliopi Petropoulou, Roger Robert Lebel, Ai Sakonju

**Affiliations:** ^1^ Department of Pediatrics Johns Hopkins Hospital Baltimore Maryland; ^2^ United States Army Medical Corps Fort Drum New York; ^3^ Child and Adolescent Health Associates Samaritan Health Systems Watertown New York; ^4^ Center for Development, Behavior, and Genetics SUNY Upstate Medical University Syracuse New York; ^5^ Department of Radiology SUNY Upstate Medical University Syracuse New York; ^6^ Department of Neurology SUNY Upstate Medical University Syracuse New York; ^7^ Present address: Department of Pediatrics Johns Hopkins Hospital Baltimore Maryland

**Keywords:** 18q deletion, myelin, myelin basic protein, ring chromosome 18

## Abstract

Myelin basic protein (MBP) contributes to peripheral and central nervous system myelin. Developmental myelinopathies exist on a clinical spectrum, but *MBP* is not included on leukodystrophy or CMT gene panels. This ring chromosome 18 case presents serial MRI and EMG/NCS, shedding light on the early clinical course of the disorder.

## Graphical abstract

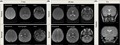

## 1 INTRODUCTION

Chromosome 18q deletions are commonly associated with hypotonia and brain MRI findings suggestive of abnormal myelination, presumably driven by haploinsufficiency of the myelin basic protein gene (*MBP*) located at Chr18q23. The *MBP* gene product is a major constituent of both peripheral and central nervous system myelin, but surprisingly few studies have investigated the consequences of distal 18q deletions for peripheral nerve conduction. We describe a 3‐year‐old girl with hypotonia, nystagmus, left ankle inversion, and mild global delay who was found to have a high‐grade mosaicism for ring chromosome 18 with resultant deletion of the distal portion of 18q. Brain MRI was consistent with almost complete absence of myelination. EMG/NCS demonstrated slowed conduction velocities in sensory and motor nerves. Despite these findings, the patient surprisingly has only mild difficulties with coordination, action tremor, and fine and gross motor tasks. These findings support both central and peripheral dysmyelination. We examine the potential role of *MBP* in this case and advocate increased utilization of EMG/NCS in developmental myelinopathies.

Ring chromosomes, although rare, usually occur as de novo events of maternal origin, in which the distal ends of the chromosome break and rejoin, resulting in partial monosomy of both regions.^1^ Of the ring chromosomes reported, r(18) is the most common, with patients generally sharing features of 18q (OMIM #601808) and 18p (OMIM #146390) deletion syndromes. The 18q deletion syndrome is variable but generally results in myelination defects, hearing loss, foot deformities, dysmorphic facies, developmental delay, failure to thrive, and hypogammaglobulinemia.^2^ Myelin basic protein gene (*MBP*) is located on the very distal end of 18q in the *Golli‐MBP* gene complex.^3^ The MBP protein makes up 30% and 15% of the central and peripheral nervous system (CNS and PNS) myelin proteins, respectively, and is important for myelin compaction.^4^ However, despite the similar role and localization of MBP to hereditary neuropathy genes, few studies have investigated peripheral nerve conduction.

## 2 CASE REPORT

The patient was the second child born to nonconsanguineous parents of Northern European descent at 37 weeks after induction of labor due to maternal hypertension. There were no abnormalities on routine nonstress tests or ultrasounds throughout the pregnancy. Initial Apgar scores were 8 and 9 at 1 minute and 5 minutes, respectively.

**Figure 1:**
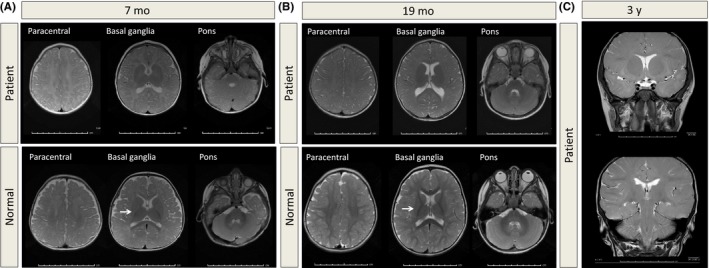
Lack of normal‐appearing white matter across development. T2‐weighted axial fast spin echo images of the patient compared to control at different ages: A, 7 mo. Lack of normal‐appearing white matter across the prerolandic (not pictured), paracentral, basal ganglia, and pons regions. B, 19 mo. Minimal myelination of the splenium and genu, but no myelination of the internal capsule and subcortical white matter. Arrows in (A) and (B) point to normal signal in the age‐matched control. C, 3 y. Minimal disorderly distribution of subcortical white matter and lack of normal myelination elsewhere

The patient had poor latch and suck‐swallow coordination. She was admitted to the hospital at 4 weeks for failure to thrive. Intestinal malrotation was discovered, and a G‐tube was placed at 3 months. The patient was exclusively fed breast milk supplemented with formula through 15 months. Ankyloglossia was surgically repaired. Seizures were suspected, but EEG was within normal limits. MRI at 7 months was consistent with complete absence of appropriate white matter myelination (Figure 1A). She underwent bilateral strabismus surgery at 1.5 years. Tonsillectomy and adenoidectomy were done for hypertrophy and sleep apnea at two years. Bilateral myringotomies were performed, and recent audiometry was within normal limits. Cardiology workup revealed no abnormalities. Endocrinology workup showed only mildly elevated TSH at 6.58 mIU/L (RR 0.3‐5 mIU/L) and free T4 at 1.51 ng/dL (RR 0.9‐1.4 ng/dL).

Family history was significant for childhood intestinal malrotation in the father. The patient's mother was diagnosed with Parry‐Romberg syndrome as a child.

The patient has been hypotonic since birth, with delayed motor milestones: sitting at 11 months, crawling at 16 months, pincer grasp at 18 months, and walking at 23 months. Language was delayed, with first words at 15 months. At 3.5 years, the patient has made substantial developmental progress with twice weekly physical, occupational, and speech therapy. The G‐tube was removed, and there are no aspiration concerns. She can now climb stairs using alternate steps and jump on two feet. She has slight left foot oversupination. She is able to keep up athletically with peers, but struggles with coordination, falling frequently. She has learned to ride a tricycle. She speaks in full sentences with prepositions but struggles with articulation. She has a preoccupation with chewing. She can recite numbers 1‐10 but cannot count the number of objects presented to her. She matches colors but does not name them. She can name shapes, holds a pen appropriately, but only scribbles. She can answer her full name and age.

On exam, the patient continues to have end gaze and up gaze nystagmus but with good visual acuity. Blue sclerae noted at birth have since resolved. Cranial nerves are grossly intact. She has a mild action tremor, which worsens with excitement. Tone is mildly decreased throughout and muscle strength is at least 4/5. Deep tendon reflexes are 2/4 and symmetrical with down‐going plantar reflexes. There is no Gowers’ sign. A sacral dimple noted earlier has resolved. She has short fifth metatarsals and syndactyly of toes 2‐3. Limbs and torso were not grossly disproportional. Head circumference is 16th percentile, weight is 17th percentile, and height is 1st percentile.

Initial karyotyping revealed 46,XX,r(18)(p11.32q22.2) with mosaicism of monosomy 18 (~7%) and trisomy for double ring chromosomes (<1%). Microarray analysis revealed a 9.775MB deletion of chromosome 18q and two adjacent duplications of 278 kbp and 646 kbp at 18q22.2. Two additional variants, a 205 kbp duplication of 2q33.1 and 244 kbp duplication of 4p16.1 are shared with her mother and likely benign. Two other known benign CNVs at 9p24.3 and 17q23.3 are shared by the mother and the child. No variants were found in her father.

Nerve conduction studies at 3 years old revealed decreased velocities in the left median sensory (40 m/s), median motor (41‐43 m/s), and ulnar motor (41‐43 m/s) nerves (age‐adjusted normal > 50 m/s). Left deep peroneal motor nerve conduction and all amplitudes were within normal limits. F wave latencies were grossly normal. Needle EMG of the left leg showed no abnormal spontaneous activity such as fibrillations or myotonic discharges; however, it did reveal large amplitude motor units consistent with a chronic neurogenic process. The mother's left median sensory and motor with F wave testing were within normal limits.

In addition to the initial MRI at 7 months, MRI was also obtained at 19 months and 3 years. At 19 months, little T2 hypointensity consistent with myelination was visible in the splenium and genu, but there continued to be undetectable myelination of the internal capsule and subcortical white matter. By 3 years, an age when normally developing children would show an adult pattern of myelination on MRI, the patient had minimal disorderly distribution of subcortical white matter (Figure 1A‐C).

## 3 DISCUSSION

**Figure 2:**
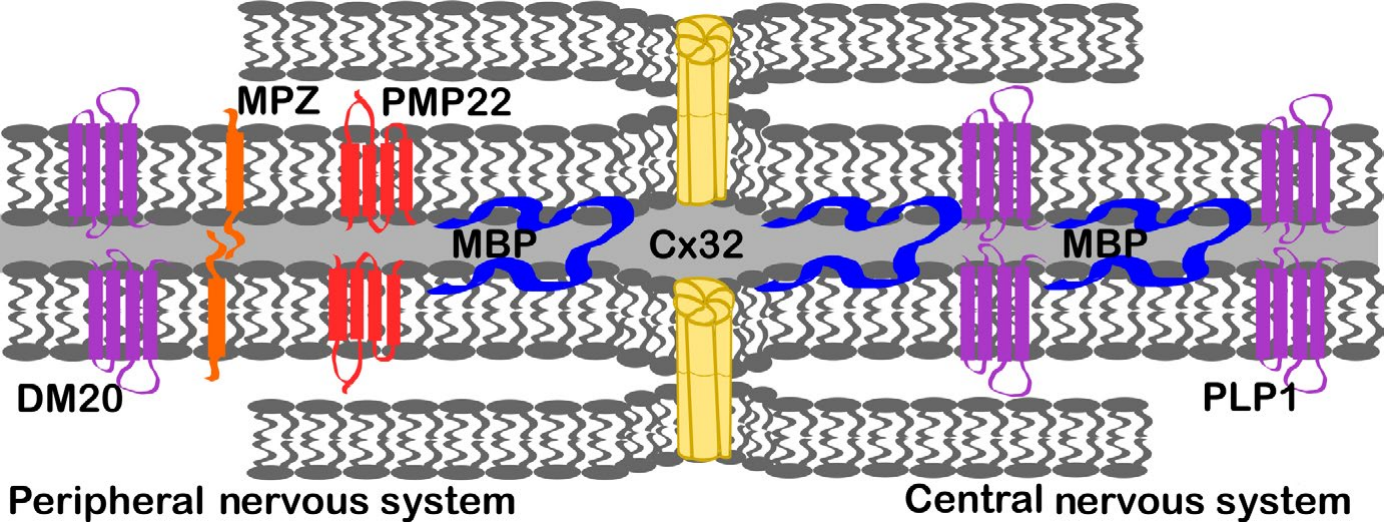
Myelin proteins schematic. The role of myelin basic protein (MBP) in myelin is shown in relation to other major myelin proteins of the central and peripheral nervous system (CNS, PNS). *PLP* is alternatively spliced to produce proteolipid protein 1 (PLP1), found predominantly in the central nervous system, and DM20, found in the peripheral nervous system. Myelin protein zero (MPZ) and peripheral myelin protein 22 (PMP22) are also relatively abundant in the PNS. Connexin 32 (Cx32) forms gap junctions across cell membranes

Myelin basic protein gene is integrated into the oligodendrocyte and Schwann cell plasma membranes, facilitating their apposition, and contributing to the major dense line of compacted myelin. Many other major myelin proteins found in this same subcellular region are associated with hereditary neuropathies, such as *PMP22, MPZ,* and *GJB1* [Figure 2; reviewed in 5. Recently, missense mutations in *PMP2* were identified in cases of CMT1A.^6^ *PLP1* is responsible for Pelizaeus‐Merzbacher Disease (OMIM #312080), an X‐linked hypomyelination syndrome with MRI reminiscent of this patient. PLP1 has two isoforms encoding proteolipid protein 1 and DM20, which are expressed predominantly in the central and peripheral nervous systems, respectively. Although Pelizaeus‐Merzbacher Disease (PMD) MRIs show dramatic changes in myelination, the few reports of EMG/NCS investigations in these patients suggests that peripheral myelination is also affected.^7^, ^8^, ^9^, ^10^

We report mildly decreased sensory and motor velocities in a patient with r(18) and *MBP* deletion, consistent with peripheral myelin pathology. Very limited accounts of EMG or NCS in similar patients exist and none at this young age. One study of a 14‐year‐old boy with 46,XY,r(18)(p11q22) reported normal peripheral nerve conduction velocity.^11^ However, a 17‐year‐old young man with r(18) was reported to have an EMG with a neurogenic pattern and prolonged distal latencies^12^ and a 36‐year‐old woman presenting with dystonia and 46,XX,del(l8)(q22.2) also had EMG/NCS suggestive of generalized sensorimotor axonal neuropathy^13^ Given the nature of genetic investigations and diagnosis in infancy or childhood, there are few longitudinal or adult r(18) or 18q‐ case reports. EMG/NCS and understanding the role of *MBP* in the peripheral, as well as the central nervous systems, will aid in predicting patient prognosis and management in cases of *MBP* deletion.

Case reports of distal 18q deletions describe diffuse MRI T2 hyperintensity consistent with delayed myelination or hypomyelination and patchy T2 hyperintensities interpreted as gliosis.^11^, ^14^ However, whether these MRI findings truly represent a lack of myelin is unclear. Postmortem neuropathology from a 25‐1/2‐year‐old male with 18q‐ syndrome who died from complications related to aortic valvular insufficiency demonstrated regions of gliosis and pallor of myelin, but central white matter and corpus callosum were noted to be well‐myelinated (.^15^ The only postmortem histopathology reported in r(18) was of a 6‐year‐old boy showing the presence of central nervous system myelination, and the authors proposed gliosis as the primary cause of MRI abnormalities^16^ It is possible that abnormal T2 signal in that case represented abnormal myelin composition and compaction instead of purely absence of myelin. Here, we describe a case with both early and longitudinal imaging, with minimal progression of myelin signal from 7 months to 3 years. The slow progression in canonical T2 hypointensity consistent with myelination might suggest delayed myelination. However, the patient's remarkably near‐normal development would suggest an under‐appreciation of the degree of myelination visible by MRI. Therefore, this case may be more consistent with dysmyelination.

To the best of our knowledge, no isolated deletions or mutations in *MBP* have been associated with a unique syndrome outside of 18q‐ and r(18) syndromes, yet *MBP* is the most likely gene in the 18q critical region affecting myelination.^2^, ^17^, ^18^, ^19^ The shiverer (shi/shi) and myelin deficient (shi^mld^/shi^mld^) mice harbor large mutations in *MBP* with autosomal recessive phenotypes consisting of tremors, followed by seizures, and early demise.^20^ In the shiverer mice, CNS myelin is almost totally absent. PNS myelin, although present, is also abnormal. Heterozygous mice express 50% *MBP* mRNA and have decreased immunohistochemical staining of MBP, but are behaviorally asymptomatic. However, increased visually evoked potential latency and increased thalamic choline signal on MR spectroscopy suggest that heterozygous loss of *MBP* does have a subclinical phenotype in these mice.^21^

In 18q‐ and r(18), deletions of multiple genes likely augment the consequence of *MBP* deletion. The galanin receptor 1 gene (*GALR1)* is immediately distal to *MBP* on 18q. Galanin is a small neuropeptide with three G‐protein coupled receptors, the first of which is encoded by *GALR1*. Galanin is secreted from oligodendrocytes and signaling promotes myelinogenesis in transgenic mice.^22^ Galanin also serves to promote oligodendrocyte proliferation, differentiation, and survival [reviewed in 23. Therefore, in addition to haploinsufficiency of *MBP*, this patient also lacks one copy of *GALR1*, potentially further decreasing or altering myelination.

No isolated cases of MBP deletion exists which may be due to a lethal effect or otherwise. DECIPHER (https://decipher.sanger.ac.uk/) predicts a high haploinsufficiency index (%HI 7.32; high rank 0%‐10%), but ExAC reports a tolerant pLI score of 0.57 (tolerant ≤ 0.1). Indeed, our patient also has a larger region of deletion which could potentially play a modifying role in her overall phenotype. There is strong evidence that cumulative effects of individually innocent copy number variants may have adverse phenotypic effects.^24^ As in our patient, auxiliary CNVs are more likely to be maternally transmitted. Although additional CNVs discovered in this patient were reported as likely benign, these CNVs may impact her clinical presentation.

In summary, this case highlights relatively early and unique MRI and EMG findings in a child with r(18) most likely attributable to *MBP* deletion. Serial MRI findings across early development and EMG demonstrate abnormal myelination. The overall phenotype may be considered as being on a spectrum with the leukodystrophies, specifically, as a milder autosomal version of Pelizaeus‐Merzbacher Disease. The expression pattern of MBP, combined with our patient's abnormal EMG/NCS and MRI, suggest that peripheral nervous system consequences of *MBP* haploinsufficiency should not be overlooked. EMG/NCS should be pursued in these patients to better inform prognosis and understanding of the natural history of the disease.

## CONFLICT OF INTEREST

The authors have no conflicts of interest to disclose.

## AUTHOR CONTRIBUTIONS

DL drafted the manuscript. All authors were involved in editing the text and provided clinical care and observations. RRL coordinated and aided in interpretation of genetic testing. KP provided MRI figures and expertise. AS conducted EMG/NCS studies and oversaw the production of the manuscript. All authors read and approved the final manuscript.
